# Cleanliness

**Published:** 1884-06

**Authors:** 


### CLEANLINESS.

OUR advances in civilization and knowledge have enabled us to correct many false theories and the customs and practices to which they gave rise ; but, unfortunately, new ones are rapidly advanced and patronized, quite as pernicious and unsound. The ancients ascribed disease to the wrath of the Gods : premature death is now considered a dispensation of Divine Providence. The practice of attempting the cure of disease by chants and incantations has given way to rational methods, but often so irrationally applied that we are left in doubt as to the value of our improvements. The uncertainty of the action of medicine and the admitted fallibility of our judgment in its administration, recalls the homely but wise old proverb, "An ounce of prevention is better than a pound of cure." Disease rarely fails to leave a permanent token of its promise to return, and however valuable curative measures may prove to be, the delicate machinery of the body but tardily resumes its wonted activity. Our conviction that a large number of diseases are preventable is every day growing stronger, and the proofs of this fact have become so numerous that it has engaged the attention of governments, and sanitary precautions are enforced by authority of law.

Filth and civilization have ever been at war. Pure air and pure water are Nature's great disinfecting agents. They come to us untaxed and unsolicited, bearing the elements of life, and convey from us the germs of disease as they exist in the products of decay. All the component parts of our bodies are in a state of constant change ; with constant growth there co-exists incessant waste and decay. These noxious and cast-off substances are nearly all soluble in the perspiration of our bodies, and must inevitably be absorbed by articles of clothing or bedding, vitiating the atmosphere with poisonous matter which may be either inhaled by the lungs or absorbed by the skin. It is a notable fact, not generally understood, that the skin plays an important part in the renovation of our bodies. Besides the worn-out material thrown off in perspiration the skin exhales about 40 cubic inches of carbonic acid gas per hour. It is estimated that one quart of fluid is thrown off from the body by perspiration every 24 hours. Can it be doubted that this excreted material, thrown off in such quantity, can fail to provoke disease ? Is it not reasonable to suppose that when brought into contact with the animal matter of our feather beds and hair mattresses, under the influence of the heat and moisture of our bodies, that chemical changes and decomposition occur? Miasmatic diseases result from vegetable decomposition; *typhoid fever from the decomposition of animal matter.* Quinine, and a judicious removal cures the former disease; while for the latter cleanliness, good food, and pure air, is both the preventive and cure. Although "Sleep is nature's sweet restorer," it is often a betrayal to our natural and ever-watchful enemy. Under the influence of sleep animal temperature falls, volition ceases, respiration becomes less frequent, and for the time resistance to disturbing causes is less vigorous and less successful. A cold draft of air produces a catarrh; marsh miasma more surely produces ague, and the absorption of decaying animal matter a low grade of fever. Awake, we take cognizance of noxious odors and unwholesome conditions; asleep, their very presence more fully blunts our sensibilities. We wash our floors with scrupulous care, whitewash our ceilings, disdain the second or third use of an unwashed towel, are shocked by a speck of rust on the butter knife, and sleep happy on beds and mattresses upon which we took our natal morning nap—quite unmindful of the fact that measles and whooping cough, scarlet fever and mumps, our grandmother's tedious typhoid fever, and our grandsire's fatal erysipelas were all suffered on that same ancient bed—the only thing of family relics left unwashed for forty years. A proper regard for cleanliness, although a weighty consideration, is by no means the most important. It is estimated that more than one million of our population are constantly sick, and of this immense number a certain proportion are the victims of contagious and infectious diseases. This fact alone constitutes a powerful plea for disinfection. The most available, and at the same time the most efficient disinfectant, is heat. It is an established fact that the contagious properties of small pox, typhoid and scarlet fevers, are completely destroyed by a temperature of from 140 to 200 deg. F. The same degree of heat will also destroy the noxious properties of excreted matter thrown off in all other diseased conditions. Blankets are preferable to quilts for beds, as they can be washed. It is well to have a thick blanket spread over the mattress, and the sheet over that, so that the mattress will be in a great measure protected from the perspiration that is constantly thrown off from the body of the sleeper, and from contamination from various diseases. Mattresses and pillows should be aired for several hours every day, and during warm weather they should be laid in the sun for an hour or two weekly, and whipped and brushed to free them from every particle of dust. Mattresses and pillows that have not been properly cared for should be made over. The covers or ticks should be washed, and the hair and feathers cleansed. The hair may be beaten with sticks, and the feathers may be put into a sheet-iron cylinder, which is made to revolve over a fire, and thus cleansed by heat.

Instead of having sleeping rooms carpeted it is better to have matting on the floors, or have the floors stained and varnished and rugs put in front of the beds. Shades for the windows are preferable to curtains, because they are easily kept clean. Many persons object to having a window and door of a sleeping room left open at night, for fear of taking cold ; but cold air is safer than vitiated air. It is only when a current of air blows directly on the back that a person takes cold ; something that will serve as a screen between the bed and the window will prevent taking cold. Avoid sleeping on the ground floor, as rooms thus located are liable to be damp, and the air not so wholesome as higher up.

In view of the many calamaties that have occurred from fire, it would seem only a reasonable precaution, and one that costs but a trifle, to provide every sleeping room above the second floor with a strong cord or rope that will reach to the ground. One end of the cord should be kept fastened to a strong staple at the side or top of the window, and the cord coiled up and hung on a peg where it can be easily thrown out as soon as the window is raised. Every person who travels much should carry a simple appliance for escaping in case of fire. One should always find out, before going to bed, the avenues of escape in case of fire, in whatever building he is to sleep.

